# Effects on microbial diversity of fermentation temperature (10°C and 20°C), long-term storage at 5°C, and subsequent warming of corn silage

**DOI:** 10.5713/ajas.18.0792

**Published:** 2019-02-14

**Authors:** Yiqin Zhou, Pascal Drouin, Carole Lafrenière

**Affiliations:** 1 University of Quebec in Abitibi-Temiscamingue, 445, boulevard de l’Universite, Rouyn-Noranda, Quebec, J9X 5E4, Canada; 2 Lallemand Specialities Inc., Milwaukee, WI 53218, USA

**Keywords:** Cold Storage, Corn, Whole-crop Silage, Denaturing Gradient Gel Electrophoresis (DGGE), Temperature

## Abstract

**Objective:**

To evaluate the effects on microbial diversity and biochemical parameters of gradually increasing temperatures, from 5°C to 25°C on corn silage which was previously fermented at ambient or low temperature.

**Methods:**

Whole-plant corn silage was fermented in vacuum bag mini-silos at either 10°C or 20°C for two months and stored at 5°C for two months. The mini-silos were then subjected to additional incubation from 5°C to 25°C in 5°C increments. Bacterial and fungal diversity was assessed by polymerase chain reaction–denaturing gradient gel electrophoresis (PCR-DGGE) profiling and biochemical analysis from mini-silos collected at each temperature.

**Results:**

A temperature of 10°C during fermentation restricted silage fermentation compared to fermentation temperature of 20°C. As storage temperature increased from 5°C to 25°C, little changes occurred in silages fermented at 20°C, in terms of most biochemical parameters as well as bacterial and fungal populations. However, a high number of enterobacteria and yeasts (4 to 5 log_10_ colony forming unit/g fresh materials) were detected at 15°C and above. PCR-DGGE profile showed that *Candida humilis* predominated the fungi flora. For silage fermented at 10°C, no significant changes were observed in most silage characteristics when temperature was increased from 5°C to 20°C. However, above 20°C, silage fermentation resumed as observed from the significantly increased number of lactic acid bacteria colonies, acetic acid content, and the rapid decline in pH and water-soluble carbohydrates concentration. DGGE results showed that *Lactobacillus buchneri* started to dominate the bacterial flora as temperature increased from 20°C to 25°C.

**Conclusion:**

Temperature during fermentation as well as temperature during storage modulates microorganism population development and fermentation patterns. Silage fermented at 20°C indicated that these silages should have lower aerobic stability at opening because of better survival of yeasts and enterobacteria.

## INTRODUCTION

Silage is produced by the fermentation by lactic acid bacteria (LAB) of humid forages and crops. In term of ensilability, whole-plant corn can be regarded as an ideal crop, because of its relatively high dry matter content (DM), low buffering capacity and adequate level of water-soluble carbohydrates (WSC) [1]. However, compared to other forage crops, corn silage is found to be more prone to aerobic spoilage when exposed to air [2]. Lactate-assimilating yeasts, and occasionally acetic acid bacteria are believed to be the primary spoilage initiators [3]. Silages with yeast counts of more than 100,000 per g are generally at a higher risk of aerobic spoilage at feed-out [3]. Also, silages with high levels of residual sugars, high concentrations of lactic acid, and low levels of volatile fatty acids (VFAs) (e.g., acetic and propionic acid) [4] are more susceptible to aerobic deterioration at feeding. This is because sugars and lactate can be readily utilized by yeasts, and an optimal concentration of VFA for conservation does inhibit growth of most yeasts [5].

Temperature affects silage fermentation [6]. In most of the studies investigating the effects of temperature on ensilage, the majority of the ensiling experiments were conducted at a moderate temperature (from 20°C to 30°C) or a fixed elevated temperature (>37°C) [7]. High temperatures are well-known for their detrimental effects on silage fermentation, resulting in poor quality and low aerobic stability silages, inducing clostridial fermentation, and heat damage [7]. Very little information is available concerning the effects of temperatures lower than 20°C on silage fermentation. Moreover, in all previous studies, evaluation has been conducted after moderately short lengths of ensiling (≤82 days). However, on many farms, silages may remain stored for longer periods of time. To our knowledge, there are no long-term studies on the effects of temperature on silage fermentation.

In the last decades, breeding has expanded corn cultivation further north following selection of hybrids adapted to cool climates. In climates similar to Eastern Canada, corn is harvested for silage in autumn when mean air temperature is around 10°C. Ensiled corn will be stored and consumed during winter and spring seasons when other fresh forages are still not available. In spring, as temperature warms up, corn silage, particularly the outer layers of the silo will be confronted to the rising temperatures.

In our previous study [8], it was found that, during 60 days of incubation, different fermentation temperatures induced distinct LAB populations during corn ensiling process, which resulted in distinct biochemical and microbiological profiles. We hypothesize that similar changes in silage profiles will also be observed with rising temperatures after an additional two months of storage at 5°C, which should simulate the temperature increase observed in spring. In this study, we simulated the fermentation and storage conditions of corn silage in Eastern Canada, and our purpose was to determine the effect of an increase of incubation temperature, from 5°C to 25°C, on whole-plant corn silage which was previously ensiled at either 10°C or 20°C for 60 days and then stored at 5°C for an additional period of two months. Biochemical properties and microbiological populations including the bacterial and fungal population diversity were determined.

## MATERIALS AND METHODS

### Silage preparation

Corn hybrid (Dekalb D26-78) was seeded in the Témiscamingue region of Québec, Canada, at a density of 74,074 plants/ha. A total of 150 kg N/ha, 70 kg P/ha, and 40 kg K/ha were applied during the growing season. After 129 days of growth, corn plants were harvested at 1/3 milk line and chopped (average particle size of 1.0 cm) with a forage harvester (New Holland 900, Belleville, PA, USA) on 20 September 2010. No silage inoculants were added. The fresh material was immediately transported to the laboratory where experimental silos were prepared. Chopped corn was used to fill 40 plastic pouches (10×16×6 mil) at a weight of 350 g/pouch [9]. Air was removed using a commercial vacuum sealer (Nel 216/219M, Hi-Tech Vacuum, Saint-Cyrille-de-Wendover, QC, Canada) according to parameters described by Johnson et al [10] with a set density of 0.490 g per cubic centimeter.

**Figure 1:**
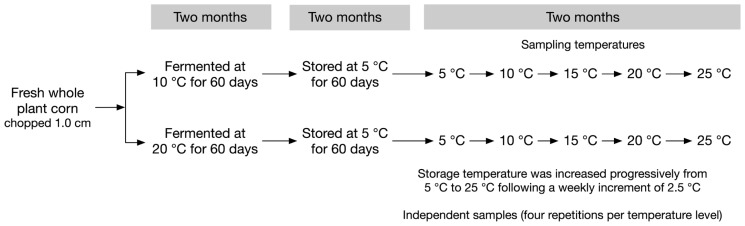
Schematic representation of the treatments applied on the chopped whole plant corn forage.

A factorial experiment with fermentation temperature (10°C and 20°C)×storage temperature (5°C, 10°C, 15°C, 20°C, and 25°C) was carried out. The 40-experimental vacuum bag mini-silos were split into two groups of 20 silos each, and incubated at either 10°C or 20°C, respectively, for two months (Figure 1). After that initial fermentation steps, all 40 mini-silos were stored at 5°C for another two months. Afterwards, storage temperature was increased progressively from 5°C to 25°C following a weekly increment of 2.5°C. Four repetitions were made for each treatment. It took two weeks for storage temperature increased from 5°C to 10°C, 10°C to 15°C, and etc. Four silos from each storage temperature treatment (5°C, 10°C, 15°C, 20°C, and 25°C) were sampled at the end of each incubation period. Thus, silos which were sampled at 5°C had been stored for four months and silos which were sampled at 25°C had been stored for six months. This represents what happened on commercial farms.

### Sampling

For each experimental silo, 20 g of silage were sampled for pH measurement, whereas 100 g of silage were sampled for the biochemical analyses. Finally, 20 g of silage were used to perform microbial enumeration and another 20 g were used for molecular microbial diversity analyses. Fresh chopped corn samples prior to ensiling were submitted to similar biochemical analyses (i.e., pH, DM, WSC, and total N) and microbial enumeration.

### Biochemical analyses

The pH was measured with a pH meter (Accumet AB15, Fisher Scientific, Toronto, ON, Canada), where duplicate samples (10.0 g) of silage were macerated with 100 mL distilled water and spun at 200 rpm for 60 min at 4°C. The DM content was determined by oven-drying for 72 h at 55°C±2°C and the dry sample was ground in a Standard Model 4 Wiley mill (Arthur H. Thomas Co., Philadelphia, PA, USA) at 1 mm. Total N was determined according to the method 7.022 of AOAC (1990), and WSC were extracted using water (100 mg of ground forage in 25 mL distilled water) and measured by the phenol sulphuric acid colorimetric method according to Dubois et al [11]. Ethanol, lactic acid, acetic acid, propionic acid, n-butyric acid, and iso-butyric acid were determined on silage water extract according to Fussell and McCalley [12]. Analyses were conducted with a gas chromatograph (Model 6850, Agilent, Mississauga, ON, Canada) equipped with a 25 m capillary column (i.d. 0.319 mm; film thickness, 0.50 μm; DB-FFAP, J & W 123-3223) and a flame ionization detector. At injection of the sample, 0.5 μL, the column temperature was set to 60°C for 1 min, then oven temperature increased to 120°C at a rate of 20°C/min, to 150°C at a rate of 15°C/min, and then to 220°C at a rate of 35°C/min and maintained for 5 min. Inlet and detector temperature were 220°C and 300°C respectively. The split ratio was 25:1. The flow rate of hydrogen which was used as carrier gas was 30 mL/min. The detector gases and their flow rate were: 30 mL/min for hydrogen, 400 mL/min for air. Peaks were identified and quantified by comparison with pure standards of acetic acid (#A38, Fisher Scientific, Canada), propionic acid (#75992-320, Anachemia, Lachine, QC, Canada), iso-butyric acid (#1754 Sigma-Aldrich; St-Louis, MO, USA), n-butyric acid (#109959, Sigma-Aldrich, USA) and ethanol (Alcools de Commerce Ltd., Boucherville, QC, Canada).

In addition, 20.0 g of silage was macerated in 200 mL of 0.1 N HCl for 60 min on a reciprocal shaker set at 200 rpm and then filtered through a Whatman #541 paper. The filtrate was used for the analyses of lactic acid and ammonia. Lactic acid was determined by a spectrophotometric method according to Taylor [13]. Ammonia was determined as described by Flipot et al [14] on an automated Kjeltec 1030 (Foss, Eden Prairie, MN, USA).

### Microbial enumeration

Each silage samples (20.0 g) were blended in a Stomacher (Seward, UK) for 2 min with 180 mL of peptone water (0.2% Bacto peptone [w/v] with 0.01% Tween 80 [w/v]), and serial dilutions were prepared with the same peptone water. Total colony forming units (CFUs) of LAB, enterobacteria and fungi (i.e., yeasts and moulds) were enumerated after incubation at 28°C for three days on plates of Rogosa Agar (Oxoid, Hampshire, UK), Violet Red Bile Glucose Agar (Oxoid, UK) and malt extract agar (MEA) (BD Difco, Sparks, MD, USA), respectively. Clostridial spores were counted on reinforced clostridial agar (Oxoid, UK) according to Jonsson [15]. Triplicates of each dilution series were made.

### Analyses of bacterial and fungal diversity

Polymerase chain reaction–denaturing gradient gel electrophoresis (PCR-DGGE) fingerprinting was used to analyze the bacterial and fungal diversity in corn silage. Total DNA was extracted from silage samples using the PowerFood Microbial DNA Isolation Kit (MoBio Laboratories, Carlsbad, CA, USA).

For bacterial diversity, we tested four primer sets and retain 357F (5′-CCT ACG GGA GGC AGC AG-3′)/517R (5′-ATT ACC GCG GCT GCT GG-3′) [16] since it produced more distinct bands from the V3 region of the 16S rDNA of bacteria from silage. A 40-bp GC clamp (5′-CGC CCG GGG CGC GCC CCG GGC GGC CCG GGG GCA CCG GGG G-3′) was attached to the 5′ end of primer 357F for DGGE analyses. PCR was carried out in a volume of 15 μL containing 1 μL of DNA template (50 ng), 1 X standard *Taq* reaction buffer, 200 μM of each deoxynucleotide, 0.3 μM of each primer and 0.025 U/μL of *Taq* DNA polymerase (*Taq* PCR Kit, New England BioLabs, Ipswich, MA, USA). PCR cycles consisted of an initial DNA denaturation at 95°C for 10 min, 30 cycles of denaturation at 93°C for 1 min, annealing at 48°C for 1 min, extension at 72°C for 1 min, and a final elongation step at 72°C for 5 min. DGGE was carried out using a DCode Universal Mutation Detection System (Bio-Rad Laboratories, Hercules, CA, USA) according to Kebli et al [17] with a modification in the range of denaturing gradient. PCR products (15 μL) were applied on 8% polyacrylamide gels (acrylamide:bis-acrylamide, 37.5:1) with a linear denaturing gradient range of 32% to 60% in 1× Tris-acetate-ethylenediaminetetra acetic acid electrophoresis buffer. Electrophoresis was performed at a constant voltage of 75 V and a temperature of 60°C for 16 h. Then the gels were stained with SYBR Gold (Invitrogen, Carlsbad, CA, USA) and visualized under UV illumination using a Molecular Imager ChemiDoc XRS System (Bio-Rad Laboratories, USA). DGGE profiles were analyzed using software GelCompar II version 6.5 (Applied Maths, Sint-Martens-Latem, Belgium). One matrix of band relative intensity was obtained. And the relative intensity of each band was calculated by dividing the intensity of the band by the sum of the intensity of all the bands within the lane.

As for the fungal diversity (i.e., yeasts and moulds), we tested five primer sets including NS1 (5′-GTA GTC ATA TGC TTG TCT C-3′)/Fung (5′-ATT CCC CGT TAC CCG TTG-3′) [18], NL1 (5′-GCC ATA TCA ATA AGC GGA GGA AAA G-3′)/LS2 (5′-ATT CCC AAA CAA CTC GAC TC-3′) [19], NL3A (5′-GAG ACC GAT AGC GAA CAA G-3′)/NL4 (5′-GGT CCG TGT TTC AAG ACG G-3′) [20], ITS1 (5′-TCC GTA GGT GAA CCT GCG G-3′)/ITS4 (5′-TCC TCC GCT TAT TGA TAT GC-3′) [21], and ITS1F (5′-TTG GTC ATT TAG AGG AAG TAA-3′)/ITS2 (5′-GCT GCG TTC TTC ATC GAT GC-3′) [17]. A 40-bp GC clamp (above) was attached to the 5′ end of primer Fung, NL1, NL4, ITS1, and ITS1F for DGGE analyses. PCR amplification failed with ITS1GC/ITS4. By comparing their DGGE profiles, primer set ITS1FGC/ITS2 produced more distinct bands and therefore was selected to amplify a fragment of 280 bp of the single sequence repeats region of fungi in silages. PCR was carried out in a volume of 15 μL containing 1 μL of DNA template (50 ng), 1 X standard *Taq* reaction buffer, 200 μM of each deoxynucleotide, 0.3 μM of each primer and 0.025 U/μL of *Taq* DNA polymerase (*Taq* PCR Kit, New England BioLabs, USA). PCR cycles consisted of an initial DNA denaturation at 95°C for 3 min, 30 cycles of denaturation at 94°C for 45 s, annealing at 55°C for 45 s, extension at 72°C for 1 min 15 s, and a final elongation step at 72°C for 5 min. DGGE was performed according to the same protocol described above, with a linear denaturing gradient range of 20% to 50%. DGGE profiles were analyzed as described above.

A protocol based on band excision-amplification was initially used to identify DGGE bands. Despite being time-consuming and laborious procedures, results of the following check-up DGGE showed that this protocol failed to extract specific bands from DGGE gels. High bacterial diversity in the silage samples probably explains this failure. Similar difficulties have also been encountered by many other DGGE users [22]. We thereafter tried to clone the PCR products of representative silage samples, and then identified individual DGGE bands by aligning the PCR amplicons of the clones with the PCR products of silage samples on same DGGE gels. This procedure required that the DNAs of silage samples were amplified using the same selected primers as described above, the PCR products were purified using the Wizard SV Gel and PCR Clean-Up System (Promega, Madison, WI, USA) and subsequently cloned using the pGEM-T Easy Vector System II (Promega, USA) according to the manufacturer’s instruction. 80 bacterial and 80 fungal positive clones were screened and streaked twice. Their plasmids were isolated using the Wizard *Plus* SV Minipreps DNA Purification System (Promega, USA). Sequence analysis was conducted with a BigDye Terminator v3.1 Cycle Sequencing Kit on the genetic analyzer 3130XL (Applied Biosystems, Foster City, CA, USA) at the Plate-forme d’Analyses Biomoléculaires (Université Laval, QC, Canada), where promoter primers Sp6 and T7 were used. The nucleotide sequences of clones were aligned using BioEdit software (version 7.1.3.0) and DNA sequence similarity searches were done via BLASTn against the GenBank database of the United States National Center for Biotechnology Information. Finally, representative clones with different sequences were re-amplified using same primers, and then aligned with the PCR amplicons of silage samples on same DGGE gels.

### Statistical analyses

For this factorial experiment (fermentation temperature [10°C and 20°C]×storage temperature [5°C, 10°C, 15°C, 20°C, and 25°C]), firstly, a two-way analysis of variance (ANOVA) with interaction was done with the biochemical and microbiological results. Interaction was found significant for several parameters. Thereafter, one-way ANOVA was performed on the biochemical and microbiological compositions of corn silages for each Fermentation Temperature. Normality of residual error was assessed using Shapiro-Wilk normality test and homogeneity of variance was verified with Fligner-Killeen test. If these two criteria were met, the data were analyzed using one-way ANOVA. The values for the ethanol concentration were log transformed in order to meet the criteria. Differences between treatment means were tested by Tukey honestly significant difference test in cases where statistical significance was observed (α = 0.05). Otherwise, mean value and standard error of the mean were presented in cases where either or both of the two assumptions were violated. Shannon diversity index were calculated based on the matrix of band relative intensity. All statistical analyses were performed using R (version 3.0.0, http://www.r-project.org).

## RESULTS

### Initial conditions of fresh corn forage

Fresh corn forage contained 309.90±2.72, 125.55±2.59, 13.06± 0.03 g/kg DM, WSC, and total N, respectively. Fresh corn forage pH was 5.85±0.03. The numbers of LAB, yeasts, enterobacteria, and moulds were 4.43±0.10, 4.95±0.04, 7.04± 0.19, and 4.38±0.05 log10 CFU/g fresh materials (FM), respectively. The number of clostridial spores was 3.18±0.20 log_10_ CFU/g FM. Overall, the biochemical and microbiological compositions of the fresh corn forage used in the present experiment was normal for whole-plant corn harvested at one-third milk line.

### Biochemical composition of corn silage

Table 1 gives the results of the effects of fermentation temperature on the biochemical composition of corn silages after two months of fermentation (20°C vs 10°C) and two months of storage at 5°C. All silages could putatively be considered well conserved since the pH was below the pHw (pH of anaerobic stability) [23]. Silages incubated at 10°C were less fermented than those at 20°C and had significantly higher pH value (4.04 vs 3.74) and residual WSC (95.84 vs 35.94 g/kg DM) (p<0.01). Silages fermented at 20°C also contained significantly (p<0.01) higher contents of lactic acid (59.24 vs 34.99 g/kg DM), ethanol (10.66 vs 2.83 g/kg DM) and NH_3_-N/total-N (5.41% vs 3.13%). In addition, no significant differences were found on DM and total N content, as well as single VFA concentration.

**Figure 2:**
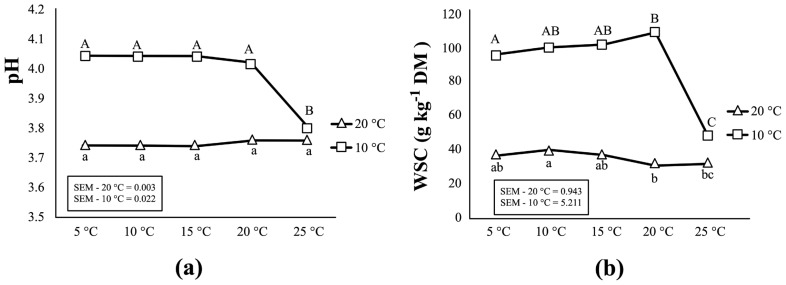
Effects of the increasing temperature (from 5°C to 25°C) during storage period on the pH (a) and WSC content (b) in corn silage initially fermented at two temperatures (10°C and 20°C). For each fermentation temperature (20°C and 10°C), values labelled with different letters within same pane are statistically different (p≤0.05).

Table 2 and Figure 2 present the biochemical changes in corn silages as storage temperature increased from 5°C to 25°C. For corn silages initially fermented at 20°C, no important changes were observed in pH and WSC content as storage temperature increased from 5°C to 25°C. No significant change (p>0.05) was found in the content of DM, total N, lactic acid, or most VFAs except acetic acid. The concentration of acetic acid, ethanol and ammonia (NH_3_-N/total-N) in these silages increase as storage temperature rose up to 25°C. On the other hand, the results from corn silages initially fermented at 10°C differ from those from silages fermented at 20°C. As storage temperature increased to 25°C, silage fermentation resumed as demonstrated by a substantial decline in WSC content (from 108.65 to 47.04 g/kg DM) and pH (from 4.02 to 3.80) (p< 0.05) (Figure 2). Simultaneously, significant increases were observed in the production of acetic acid and ammonia at 25°C (p<0.001). A biological trend was also observed for an increase in propionic acid as the temperature increase (p = 0.063).

### Microbial counts of corn silage

Microbial enumeration was made on corn silage (Table 3) after each silo openings with increasing temperature. Regarding the effects of initial fermentation temperature (20°C vs 10°C), significant higher LAB counts were detected at 20°C than at 10°C (7.85 vs 5.80 log_10_ CFU/g FM) (p<0.001) (Table 1). For silages initially fermented at 20°C, LAB counts slightly increase as storage temperature increased from 20°C to 25°C. Undesirable microbes (i.e., enterobacteria and yeasts) started to appear at 15°C and above. On the contrary, in silages initially fermented at 10°C, LAB counts increased sharply as storage temperature reached 20°C and 25°C (p<0.05), and neither enterobacteria nor yeasts were detected (<2.00 log_10_ CFU/g FM).

No moulds were detected in all silage samples (<2.00 log_10_ CFU/g FM). Number of clostridial spores was not affected by neither initial fermentation temperatures (20°C vs 10°C) (Table 1) nor following temperature increases from 5°C to 25°C during the storage period. Moreover, the numbers of spores in corn silage were similar to those in corn forage prior to ensiling. These findings indicated that the growth of *Clostridium* was inhibited, and no germination of clostridial spores took place during the entire experimental period.

### Bacterial diversity of corn silage

**Figure 3:**
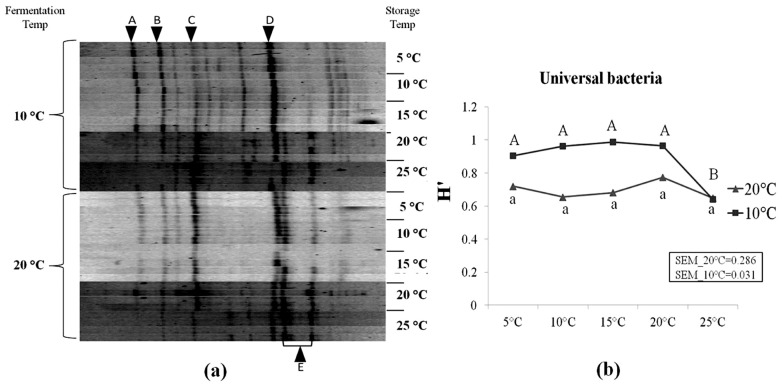
Denaturing gradient gel electrophoresis profiles of universal bacteria in corn silage (a) and their corresponding Shannon diversity index (H′) (b). Labeled bands with letter “A” to “E” were allotted to the following species: A, *Weissella koreensis*; B, *Leuconostoc citreum*; C, *Lactobacillus brevis*; D, *Chryseobacterium sp.*; E, *Lactobacillus buchneri*. Four repetitions of every experimental treatment are included.

LAB species represented the major bacterial population, in particular species such as *Lactobacillus buchneri* (*Lb. buchneri*), *Lactobacillus brevis* (*Lb brevis*), *Weissella koreensis*, and *Leuconostoc citreum* (Table 4). Other LAB species such as *Lactobacillus oryzae* and *Pediococcus parvulus* were also found. Enterobacteria such as *Pantoea agglomerans* were detected. Our results indicated that, a clone identified of the genus *Chryseobacterium*, was reported for the first time in corn silage in rather high occurrence as shown by its high relative intensity in Figure 3. Figure 3 presents the DGGE profiles of universal bacteria in corn silages and the corresponding Shannon diversity index (H′). Higher diversity was observed in corn silages initially fermented at 10°C compared to 20°C. H′ of silages initially fermented at 20°C was not affected by storage temperatures (p>0.05). However, in silages which were fermented at 10°C, a significant decrease in H′ was observed as storage temperature increased to 25°C (p<0.05). At 25°C, the H′ value of both initial fermentation treatments reached the same value. In addition, six operational taxonomic units (OTUs) in the DGGE profiles were aligned to bacteria clones. It was noticed that the relative intensity of *Lb. buchneri* (OTU E) tended to increase at higher storage temperatures, particularly in silage samples which were initially fermented at 10°C.

### Fungal diversity of corn silage

**Figure 4:**
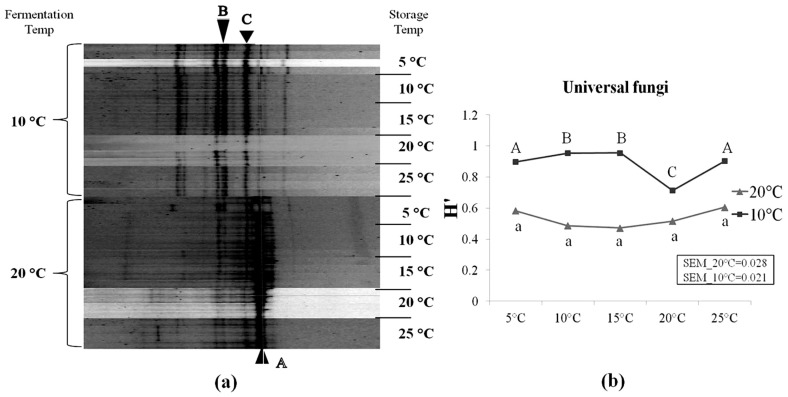
Denaturing gradient gel electrophoresis profiles of universal fungi in corn silage (a) and their corresponding Shannon diversity index (H′) (b). Labeled bands with letter “A” to “C” were allotted to the following species: A, *Candida humilis*; B, *Uncultured Davidiella*; C, Uncultured *Basidiomycota*. Four repetitions of every experimental treatment are included.

As shown in Table 5, several species of mould flora were detected in corn silage samples using the cultural-independent approach. However, the microbial counts showed that moulds were under the detection level on the MEA medium (Table 3). In addition, some mould spores may also be present in silages, but cannot germinate and grow on the MEA plates due to the unsuitable lab conditions. This suggests that culturable fungi species on the MEA medium accounts for only a small portion of whole fungal community in silages, and the method of enumeration of microbial colonies on this medium underestimate the true number of moulds present in silage. Following sequencing, several mould genera were detected, including *Oidiodendron*, *Cladosporium*, *Fusarium*, *Davidiella*, *Basidiomycota*, *Tremellales*, and *Alternaria*. It was also observed that the yeast species *Candida humilis* (*C. humilis*) were frequently detected in our samples. The DGGE profiles of silages fermented at 10°C differed from those fermented at 20°C (Figure 4). Higher diversity level of fungi (i.e., yeasts and moulds) was observed in silages fermented at 10°C compared to 20°C. As storage temperature increased from 5°C to 25°C, no important changes were observed within neither of the two fermentation temperatures. This can also be seen along the difference in H′ between both temperatures (Figure 4).

## DISCUSSION

### Effects of fermentation temperature (10°C vs 20°C) on the fermentation of whole-plant corn silage

The results from the current study are generally consistent with our earlier findings [8] that low fermentation temperature (10°C vs 20°C) restricts silage fermentation, resulting in higher pH, more residual WSC, less lactic acid production, lower levels of ethanol and ammonia, as well as lower numbers of LAB (p<0.05) (Table 1).

### Corn silages initially fermented at 20°C did not stabilize as storage temperature increased from 5°C to 25°C

After an additional two months of storage at 5°C, a highly acidic environment was reached (pH = 3.74) in corn silage initially fermented at 20°C. Little changes occurred as storage temperature increased from 5°C to 25°C. Basically, the increasing storage temperature from 5°C to 25°C did not affect much the fermentation profiles, in terms of most biochemical parameters (i.e., pH, WSC, DM, total-N, lactic acid and most VFAs) (Figure 2, Table 2), as well as for the bacterial and fungal populations (Figures 3, 4). The number of LAB colonies as determined from plate counts stayed rather stable, although a statistically significant increase was observed at 25°C (Table 3). In addition, DGGE analysis results showed that the LAB flora in corn silage consisted of mainly heterofermentative species, such as *Lb. buchneri*, *Lb. brevis*, *Leuconostoc citreum*, and *Weissella koreensis* (Figure 3). This is in agreement with our results [8] as well as many previous studies that heterofermentative LAB species usually predominate in the latter stages of ensilage [24].

It should be noticed that, as incubation temperatures rose up to 15°C, a fairly high number of enterobacteria and yeasts (4 to 5 log_10_ CFU/g FM) were detected. Enterobacteria and yeasts are facultative anaerobes. Under anaerobic conditions, enterobacteria ferment available sugars producing mainly acetic acid, with lesser quantities of ethanol, formic acid, 2,3-butanediol and CO_2_. Some enterobacteria can also deaminate amino acids to ammonia. Yeasts ferment sugars to mainly ethanol and CO_2_ along with small amounts of other alcohols and VFAs. In addition, the activity of plant proteases was reported to increase as temperature increases, which results in greater protein breakdown and ammonia accumulation in silages [25]. This could explain the significantly higher contents of acetic acid and ammonia (p<0.05) at higher storage temperatures, as well as the significantly higher concentration of ethanol (p<0.05) (Table 2; fermentation temperature 20°C). Furthermore, our DGGE results showed that the yeast *C. humilis* predominated the fungi flora in these silages (Figure 4). *Candida* spp. are commonly detected in ensiled samples, and *C. humilis* has been reported to predominate in whole-plant corn silage using classical plate counts techniques [26,27], but also following operon-based metasequencing [28]. Most *Candida* spp. can readily assimilate lactate for their metabolism under aerobic conditions [29]. Therefore, these corn silages which were initially ensiled at 20°C might be prone to aerobic deterioration, as it was reported that yeast number higher than 10^5^ CFU per gram is considered a threshold level to induce aerobic deterioration at feed-out [30].

### Corn silage initially fermented at 10°C resumed fermentation as storage temperature increased from 5°C to 25°C

As storage temperature increased from 5°C to 20°C, no significant changes were observed in most silage characteristics, including pH, WSC content, lactic acid, ethanol and most microbial counts (Tables 2, 3; Figure 2). But, a significant increase in the number of LAB colonies was observed at 20°C and 25°C (Table 3). Results of the bacterial diversity measurements further showed that, as temperatures reached 20°C and 25°C, two heterofermentative species, *Lb. buchneri* and *Lb. brevis*, became the dominant species of the LAB flora in these silages (Figure 3). As a consequence, the content of acetic acid in these silages increased considerably (by 71.5%) at 25°C, and this was in accordance with the rapid decline in pH and WSC concentration (Figure 2). In addition, ammonia concentration also increased as temperature rose up to 25°C putatively due to the increased in proteolysis from plant-based enzymes. All above changes clearly indicated that silage fermentation resumed as temperatures increased. Indeed, it has been observed in cool climates that silage fermentation could restart as weather warms up, in particular for the less fermented silages due to low temperature at ensiling [7].

Yeasts were under the detection level in these silages (<2.00 log_10_ CFU/g FM) (Table 3), and DGGE results showed that the lactate-assimilating species *C. humilis* represented only a small fraction of the fungi flora (Figure 4).

As storage temperature increased to 20°C and 25°C, *Lb. buchneri*, started to prevail (Figure 3). The metabolism of *Lb. buchneri* [31] and the merits of inoculating with *Lb. buchneri* to improve silage aerobic stability have been well studied [32–34]. Hence, due to the increased activity of the heterofermentative *Lb. buchneri*, more acetic acid was produced (p<0.001) along with small increased of propionic acid (p = 0.063), ensuring the absence of undesirable yeasts in silage. The increase in lactic acid observed during the last incubation phase, although not significant, could be due to low activity of homofermentative strains. A combination of the low incubation temperature and higher pH (10°C incubation series) could protect the cells homeostasis [35].

### *Chryseobacterium* sp

The occurrence of DGGE OTU (Figure 3) and clones (Table 4) belonging to *Chryseobacterium* sp. is puzzling since this bacterium is rarely mentioned in silage, neither do other members of this family. The only occurrence was very recent in an operon-based metasequencing analysis of grass silage [36]. They reported that 3% of the diversity was related to *Flavobacterium* and *Chryseobacterium*, and nearly 10% for the Flavobacteria. Ridwan et al [37] also reported the identification of *Chryseobacterium* following T-RFLP of grass-legumes silages. Another operon-based metasequencing projects observed this genus in around 5% of the OTU from fresh whole plant corn and 2% of the OTU from non-inoculated ensiled samples (Drouin, personal communication). *Chryseobacterium* is a ubiquitous bacterium belonging to the Flavobacteriaceae family. Some bacteria from this family are often used to produce extracellular enzymes as silage additives, but this does not explain their presence since no inoculant were used. Although they are frequently observed in soils, they were also isolated from decayed plants [38]. Since some of the corn tissues are in senescence, this bacterium could actively colonize decaying leaves or teasel. By doing so, they would become part of the epiphytic microflora of corn. Residual DNA would then have been amplified by PCR. Presence of the corresponding OTU on fresh material (results not presented) confirmed this hypothesis. It was also observed that the intensity of this OTU was higher following ensiling at 10°C than at 20°C, and it diminished as storage temperature increased. Degradation of dead microbial cells and residual DNA from this organism could be more important as the temperature increased. The occurrence of this organism on corn plants prior to ensiling could affect silage quality in relation with suboptimal fermentation parameters in early stages of ensilage.

To conclude, this experiment simulated what happens to commercial silages as storage temperature gradually increases in spring time. Ambient temperature at ensiling affects the fermentation characteristics of whole-plant corn silage during long-term storage. Previously, it has been concluded in many short-term silage studies that moderate temperatures (between 20°C and 30°C) are more favourable for ensilage and result in better quality silage. However, in this long-term study, we showed that it was true with many fermentation parameters but might not be so in terms of the survival of spoilage microbes in silage (such as yeasts and enterobacteria). This partially explains why well-fermented silages are sometimes observed to be prone to aerobic deterioration. On the contrary, although low temperatures at ensiling restrict silage fermentation, fermentation could resume following warm-up weather. Moreover, the survival of yeasts in these silages is lower than in silages made at warmer temperature at ensiling. In practice, these observations allowed us to recommend that producers feed silages which were initially ensiled at warmer temperature earlier and quickly during winter or early spring time, in order to reduce aerobic spoilage. Moreover, for silages which were ensiled at low temperatures, it is suggested to add inoculants, such as *Lb. buchneri*, to ensure silage aerobic stability as ambient temperature warms up, particularly if the silage is used for summer feeding.

## Figures and Tables

**Table 1:** Effects of fermentation temperature (10°C vs 20°C) on the biochemical composition of corn silage (g/kg DM) and microbial counts

Fermentation temperature	pH	DM (g/kg FM)	WSC	Total N	Lactic acid	Acetic acid	Ethanol	Propionic acid	n-Butyric acid	iso-Butyricacid (%)	NH_3_-N/total N	LAB1)	Clostridia spores
	
			----------------------------------------- g/kg DM -------------------------------------------									Log_10_ CFU/g FM	
20°C	3.74	299.4	35.94	13.78	59.24	80.01	10.66	1.17	0.53	0.28	5.41	7.85	2.80
10°C	4.04	308.3	95.84	13.80	34.99	78.41	2.83	0.95	0.45	0.23	3.13	5.80	3.12
SEM	0.056	2.639	11.386	0.102	5.582	0.637	1.590	0.282	0.053	0.088	0.442	0.389	0.154
p2)	***	NS	***	NS	**	NS	***	NS	NS	NS	***	***	NS

DM, dry matter; FM, fresh materials; WSC, water-soluble carbohydrates; LAB, lactic acid bacteria; CFU, colony forming units; SEM, standard error of the mean; NS, not significant.

1) Microbial enumeration was done after two months of fermentation at each temperature and another two months of storage at 5°C. Enterobacteria and yeasts and molds count was under detection level (<2 log_10_) for both fermentation conditions.

2) Values were declared statistically different (** and *** at p≤0.01 and 0.001).

**Table 2:** Effects of the increasing temperature during storage period on the chemical composition of corn silage initially fermented at two different temperatures (g/kg DM)

Storage temperature	Total N	DM (g/kg FM)	Lactic acid	Acetic acid	Ethanol	NH_3_-N/total N (% total-N)	Propionic acid	n-Butyric acid	iso-Butyric acid
Fermentation temperature (20°C)									
5°C	13.78	299.4	59.24	80.01^ab^ 1)	10.66^b^	5.41^a^	1.17	0.53	0.28
10°C	13.87	307.9	55.92	70.64^a^	7.50^c^	5.35^a^	0.94	1.32	0.07
15°C	13.86	306.9	59.01	93.22^bc^	8.10^c^	5.42^a^	0.90	1.64	0.10
20°C	13.53	303.9	50.98	107.63^c^	13.45^a^	5.63^ab^	0.76	1.02	0.13
25°C	13.59	306.6	61.33	107.86^c^	15.25^a^	6.16^b^	1.21	1.85	0.28
SEM	0.080	1.25	1.912	4.081	0.895	0.086	0.144	0.208	0.045
Effects of increasing temperature	NS	NS	NS	***	*	**	NS	NS	NS
Fermentation temperature (10°C)									
5°C	13.80	308.3	34.99	78.41^A^	2.83	3.13^A^	0.95	0.45	0.23
10°C	13.63	314.0	40.49	80.96^A^	2.79	3.40^A^	1.10	0.22	0.19
15°C	13.70	311.3	36.08	80.17^A^	3.16	3.44^A^	1.07	0.21	0.17
20°C	13.68	304.8	44.39	81.16^A^	2.52	3.55^A^	1.41	0.54	0.36
25°C	13.97	305.2	45.41	139.17^B^	4.08	5.34^B^	1.27	0.56	0.30
SEM	0.089	1.60	1.531	5.569	0.213	0.182	0.054	0.059	0.039
Effects of increasing temperature	NS	NS	NS	***	NS	***	0.063	NS	NS

The data of ethanol was log transformed for the verification of the two assumptions before analysis of variance analysis.

DM, dry matter; FM, fresh materials; SEM, standard error of the mean; NS, not significant.

1) For each fermentation temperature (20°C and 10°C), values with different letters within the same column are statistically different (*, ** and *** at p≤0.05, 0.01 and 0.001).

**Table 3:** Effects of increasing temperature during storage period on the microbiological composition of corn silage initially fermented at two different temperatures, 10°C and 20°C

Storage temperature	Culture media ((log_10_ CFU/g FM)				

	LAB	Enterobacteria	Yeasts	Moulds	Clostridia spores
Fermentation temperature (20°C) 1)					
5°C	7.85^a^ 2)	ND3)	ND	ND	2.80
10°C	7.82^a^	ND	ND	ND	3.21
15°C	7.67^a^	4.02±0.07	3.93±0.08	ND	3.52
20°C	7.81^a^	4.71±0.47	4.85±1.21	ND	3.43
25°C	8.19^b^	4.78±0.10	4.44±1.11	ND	3.68
SEM d)	0.049	0.517	0.504		0.108
Effects of increasing temperature	**4)				NS
Fermentation temperature (10°C)					
15°C	5.80^A^	ND	ND	ND	3.12
10°C	5.85^A^	ND	ND	ND	3.37
15°C	5.85^A^	ND	ND	ND	3.28
20°C	7.43^B^	ND	ND	ND	3.54
25°C	8.54^C^	ND	ND	ND	3.58
SEM	0.255				0.061
Effects of increasing temperature	***				NS

CFU, colony forming units; FM, fresh materials; LAB, lactic acid bacteria; SEM, standard error of the mean.

1) For each fermentation temperature (20°C and 10°C), values with different letters within the same column are statistically different (p≤0.05).

2) Standard error included since analysis of variance assumptions not respected for those parameters.

3) ND, not detected (<2.00 log_10_ CFU/g FM).

4) NS, not significant; ** and ***: significant at p≤0.01 and 0.001.

**Table 4:** Putative identity of the 80 bacterial clones obtained from polymerase chain reaction amplification of silage samples

Number of clones sequenced	Most closely related bacterial sequence	GenBank accession No. of related sequence	Homology (%)	Environment from which related sequence was isolated
20E1)	*Lactobacillus buchneri*	JQ249065.1	100	Fermented cucumber (USA)
12C	*Lactobacillus brevis*	KC713915.1	100	Fermented bamboo shoot (India)
5D	*Chryseobacterium sp.*	AB461706.1	100	Stems of field-grown soybeans (Japan)
4A	*Weissella koreensis*	NR075058.1	100	Kimchi (USA)
1B	*Leuconostoc citreum*	KC417025.1	99	Wheat flours (Italy)
8	Uncultured bacterium isolate	JX183833.1	98	Jejunum, ileum and cecum of weaned piglets (China)
6	*Pantoea agglomerans*	KC355300.1	99	Pepper (South Korea)
2	*Lactobacillus oryzae*	AB731661.1	99	Fermented rice grain (Japan)
2	*Pediococcus parvulus*	AB601176.1	100	Italian ryegrass silage (Japan)
1	*Stenotrophomonas maltophilia*	KC764984.1	100	Tobacco rhizosphere soils (China)

OTUs, operational taxonomic units.

1) Clones labeled with letter “A” to “E” correspond to the marked out OTUs of Figure 3.

**Table 5:** Putative identity of the 80 fungal clones obtained following polymerase chain reaction amplifications for silage samples

Number of clones sequenced	Most closely related fungal sequence	GenBank accession No. of related sequence	Homology (%)	Environment from which related sequence was isolated
23A1)	*Candida humilis*	AY493349.1	98	Natural tequila fermentation
8	Uncultured *Oidiodendron*	JF796748.1	99	Oil pumpkin flower (Austria)
7	Uncultured fungus clone	FJ757776.1	100	Quercus macrocarpa phyllosphere (USA)
6	Uncultured *Cladosporium*	KC143740.1	100	Human stool (France)
6	Uncultured *Fusarium*	HE977545.1	99	Soil (UK)
5B	Uncultured *Davidiella*	JX448366.1	100	Agarwood (India)
4	Uncultured fungus clone	FJ757067.1	100	Quercus macrocarpa phyllosphere (USA)
3C	Uncultured *Basidiomycota*	HE977542.1	100	Soil (UK)
2	Uncultured fungus clone	JN906946.1	100	European beech (Fagus sylvatica) phyllosphere (France)
2	Uncultured fungus clone	FJ758346.1	100	Quercus macrocarpa phyllosphere (USA)
2	Uncultured *Alternaria*	JQ346916.1	100	Roots of herbs (endophyte) (China)

OTUs, operational taxonomic units.

1) Clones labeled with a letter “A” to “C” correspond to the marked out OTUs in Figure 3. Five other single uncultured fungus were also detected and cloning and/or sequence analysis of 7 fungus clones failed.
